# Predicting Reoffending Using the Structured Assessment of Violence Risk in Youth (SAVRY): A 5-Year Follow-Up Study of Male Juvenile Offenders in Hunan Province, China

**DOI:** 10.1371/journal.pone.0169251

**Published:** 2017-01-11

**Authors:** Jiansong Zhou, Katrina Witt, Xia Cao, Chen Chen, Xiaoping Wang

**Affiliations:** 1 Department of Psychiatry & Mental Health Institute of the Second Xiangya Hospital, Central South University, Changsha, Hunan, China; 2 National Clinical Research Center on Mental Disorders & National Technology Institute on Mental Disorders, Hunan Key Laboratory of Psychiatry and Mental Health, Changsha, Hunan, China; 3 Population Health, Turning Point, Eastern Clinical School, Monash University, Clayton, Victoria, Australia; 4 Department of Health Management Center, The Third Xiangya Hospital, Central South University, Changsha, Hunan Province, China; University of Texas Health Science Center at San Antonio Cancer Therapy and Research Center at Houston, UNITED STATES

## Abstract

**Background:**

Juvenile violent offending is a serious worldwide public health issue.

**Objective:**

The study examined whether the Structured Assessment of Violence Risk in Youth (SAVRY) can be used to predict violent reoffending in Chinese male juvenile offenders, and to determine which risk/protective domains (items) are associated with violent recidivism.

**Methods:**

A total of 246 male juvenile offenders were recruited. SAVRY domains were scored by trained raters based on file review and interviews with participants and their legal guardians. Information on further arrests, charges, or convictions for violent offences were collected from police records over a five year follow-up.

**Results:**

Over the course of the five year follow-up periods, 63 (25.6%) juvenile offenders were re-arrested for a further violent reoffence. Receiver Operating Characteristic (ROC) analyses showed Areas Under the Curve (AUCs) ranging from 0.60 to 0.68 for the SAVRY total, risk and protective score domains. Univariate logistic regression analysis showed that 7 of the 30 SAVRY items were significantly associated with reoffending; explaining 36.2% of the variance. Backward stepwise multiple logistic regression analysis showed the independently predictive items were items 2 (‘history of non-violent offending’), 17 (‘negative attitudes’), 18 (‘risk-taking/impulsivity’), and 20 (‘anger management problems’). Together these four items explained 25.0% of the variance in reoffending.

**Conclusions:**

The results suggested that the SAVRY can be meaningfully used to inform the development and evaluation of effective violence risk assessment and management approaches for male juvenile offenders detained in a Youth Detention Center in Hunan province, China.

## Introduction

Violence is currently one of the top 20 causes of disability life-adjusted years lost worldwide [1]; costing the United States economy alone $37 billion in health care and productivity costs each year [2]. Internationally, rates of violent and sexual recidivism have declined significantly in juvenile offending populations over recent years in a number of jurisdictions [3]. In the United States, for example, juvenile arrest rates for violent offences have declined by as much as 55.0% between 1994 and 2014 [4] and, for sexually violent offences, by as much as 73.0% over this same time period [3].

In China, however, recent data suggests there has been an increase in general criminal recidivism over the last decade [5]. Additionally, there is some data to suggest youth offending has increasingly shifted towards more seriously violent offences [6], including murder, armed robbery, rape, and assault [7]. According to data from the China Statistical Yearbook, moreover, around 20,000 juvenile offenders have been detained in Youth Detention Centers (YDCs) each year from 2000 for predominately violent offences [8].

The assessment of violent recidivism risk has therefore become central to juvenile criminal justice decision-making process in China in recent years. The 2013 National Mental Health Law and the recent changes to the Criminal Procedure Code, for example, both emphasise the centrality of risk assessment and management protocols. In particular, these changes now ensure that, in China, each juvenile offender receives a risk assessment to assess their future risk of reoffending and that they, and their families, are provided with advice on mitigating this risk on release.

Although a number of standardised risk screening and assessment instruments have been developed to assess violence risk within juvenile offender populations, the majority of these instruments have been developed and validated in Western samples [9]. As a consequence of their low predictive validity in Asian populations [9], and the fact that different risk factors appear to have salience for the prediction of violence risk in Asian as compared to European-American populations [10], forensic mental health specialists in China are less likely than their European or American counterparts to use these instruments [11]. Instead, Chinese forensic mental health specialists have tended to use unstructured clinical judgment when assessing violence risk [12,13]. Although the use of this approach is not generally recommended in the West, owing to problems with reliability and predictive validity, we have previously shown that a number of standardised actuarial and structured professional judgement instruments are also associated with poor predictive validity when used in Chinese samples [9]. We therefore concluded that further evaluations of the reliability and validity of these instruments are required before they can be recommended for widespread use in China.

Although the Structured Assessment for Violence Risk in Youth (SAVRY) is one of the most widely used instrument for the assessment of violence risk in juvenile offenders worldwide, the predictive validity of this instrument is yet to be demonstrated in Chinese juvenile offenders. A recent study from Singapore, however, found that none of the SAVRY risk domains were predictive of violent recidivism, suggesting no particular advantage for the SAVRY in Asian samples over other instruments designed for the assessment of general, rather than violent, recidivism [14]. We therefore sought to investigate whether the SAVRY can be used to predict violent reoffending in Chinese juvenile offenders and, further, which of the risk/protective domains and individual items are most strongly associated with violent recidivism in this group.

## Materials and Methods

This study forms part of a larger investigation into the assessment, prevention, and treatment of adolescent male offenders in the China. The procedure of this larger study is reported in greater detail in a related publication [15].

### Participants

The sample comprised 246 male juvenile offenders between 15 and 18 years of age consecutively detained in a Youth Detention Center (YDC) in Changsha, Hunan Province, China between August and November, 2008. Over half of the offenders in this sample (n = 163, 66.3%) had been convicted of violent offences, including: robbery (n = 101; 41.1%), assault (n = 32; 13.0%), sexual violence (n = 17; 6.9%), and homicide (n = 13; 5.3%). Around one-in-three (n = 83; 33.7%) had been convicted of a lesser offence, including theft where there was an element of threatened violence, such as use of a weapon or carrying a weapon in hand. All offenders were sentenced to no more than three years’ detention; the average number of months in detention was 10.9 months (SD = 9.9, range = 1 to 36 months). After their release from the YDC, these juvenile offenders were paroled and subjected to supervision orders requiring frequently (i.e., monthly) contact with their local police office. No offender included in this sample received any further interventions during this time.

Each province in China has one YDC in which all males between 15 and 18 years of age convicted of violent offences are detained following sentencing. Offenders detained in these YDCs are therefore representative of the population of sentenced, violent male juvenile offenders residing in Hunan Province, which has a total population of 67.47 million as at 2014 [8]. Further information on the characteristics of this YDC, however, is reported in a related publication [15].

The average age of the offenders at referral to the study was 16.7 years (SD = 1.0, range = 15–17). The average length of education was 7.3 years (SD = 1.8, range = 1–10 years). One tenth (n = 25, 10.1%) of the current sample had a history of delinquency behaviour contact with police predating the violent offense that resulted in detention in the YDC.

### The Structured Assessment of Violence Risk in Youth (SAVRY)

The Structured Assessment of Violence Risk in Youth (SAVRY), based on the structured professional judgement (SPJ) model, is designed to assist in the assessment and management of juveniles between the ages of 12 to 18 years who have either offended violently or are considered at risk of doing so on the basis of certain risk factors derived from epidemiological research with Western samples [16,17].

The SAVRY comprises 24 items organised into three risk domains: Historical (10 items), Social/Contextual (6 items), and Individual/Clinical (8 items). Each item has a three level rating structure ranging from “0 = low” to “1 = moderate” to “2 = high”. The SAVRY also includes 6 protective items scored as either 0 = absent or 1 = present. These items include: prosocial involvement, strong social support, strong attachment and bonds, positive attitude toward intervention and authority, strong commitment to school, and resilient personality traits.

The SAVRY Total Score is derived by summing the individual item scores on the Historical, Social/Contextual, and Individual/Clinical risk domains as well as the 6 individual protective items. The SAVRY Risk Total Score is derived by summing the items from the risk domains only whilst the SAVRY Protective Total Score is derived by summing scores on the 6 individual protective items only. For clinical purposes, raters would then derive a Summary Risk Rating of low, moderate, or high risk of violence after giving consideration to the nature of the risk factors and protective factors present in each individual case. For research purposes, however, we calculated only the SAVRY Total, Risk, and Protective Scores. We also calculated individual domain (i.e., Historical, Social/Contextual, and Individual/Clinical) and individual item scores.

### Procedure

This study was approved by the Biomedical Ethics Board of the Second Xiangya Hospital, Central South University, Hunan province (2007038), China. Written informed consent was required both from the participants themselves and from their legal guardian prior to participation. Prior to the commencement of this study, eligible participants and their legal guardians were given both oral and written information relating to the aims of this study. Participants were reminded that their decision to participate was voluntary, and that refusal to provide consent would not affect either their judicial status or their length of detention in the YDC. All discussions relating to participation were conducted in a private area of the YDC. No compensation was offered for participation.

Items on the SAVRY were scored by trained raters based on file review and interviews with participants and their legal guardians by two trained forensic psychiatric fellows. These raters were trained in the user of the SAVRY by the corresponding author, a psychiatrist with 6 years’ experience assessing violence risk in juvenile offenders in China. The resulting interrater reliability (kappa) was above 0.81 for each of the risk and protective items assessed by the SAVRY, indicating the strength of agreement between raters was very good [18].

Information on further arrests, charges, or convictions for violent offences were collected between October and November, 2013 –i.e., following an average follow-up period of 5 years—from **local** official police records. For the purposes of this study, violent offences were defined according to the definition contained in the SAVRY manual (i.e., “an act of battery of physical violence sufficiently severe to cause injury to another person or persons, regardless of whether this injury occurs…or a threat made with a weapon in hand”)[17]. Violent offences therefore could include: homicide, attempted homicide, assault, robbery, causing bodily harm, making threats to kill or harm, sexual violence and unlawful use of a weapon.

### Statistical analyses

Chi-square tests were used to compare categorical variables and, for continuous variables, either the t-test of the Mann-Whitney U test was used as appropriate. Specifically, as the Kolmogorov-Smirnov and Shapiro-Wilk tests suggested that age, duration of education, and father’s and mother’s duration of education were not normally distributed (p<0.001), the Mann-Whitney U test was used in preference to the t-test.

Receiver Operating Characteristic (ROC) analyses were also used to determine whether the SAVRY Total Score, SAVRY Risk Score, SAVRY Protective Score, and individual domain (i.e., Historical, Social/Contextual, and Individual/Clinical) scores were predictive of violent reoffending over the five year follow-up period. Univariate logistic regression analyses were also performed to investigate the association of the individual SAVRY risk and protective items with violent reoffending over the five year follow-up period. Specifically, backward multiple logistic regression analyses were used to identify the independent contribution of these individual SAVRY items in predicting violent reoffending. Items with p values <0.10 in the univariate analyses were included in the multiple logistic regression model. Only those associated with a p value of <0.05 were included in the final model, however. Adjusted Odds ratios (ORs) and their associated 95% confidence intervals (CIs) were reported in the text. All analyses were conducted using SPSS for Windows, version 21.0 [19].

## Results

Following the conclusion of the five year follow-up period, of the 246 young male offenders, 63 (25.6%) had been rearrested for a further violent offence according to official police records. There were no significant differences between the reoffending and no reoffending groups with regard to age, duration of education, father’s and mother’s duration of education, parental marital status or family income at baseline (Table 1; all p values >0.05).

**Table 1:** Descriptive statistics on the demographics characteristics of reoffending and no reoffending youth offenders on baseline (*n* = 246).

	No reoffending (n = 183)		Reoffending (n = 63)		*Z*	*df*	*p*-value
	M	SD	M	SD			
Age (years)	16.8	(0.9)	16.6	(1.0)	1.6		0.11
Education (years)	7.4	(1.8)	7.3	(2.7)	0.1		0.92
Father’s duration of education (years)	7.5	3.0	7.3	3.0	0.2		0.84
Mother’s duration of education (years)	6.3	3.5	6.3	3.7	0.4		0.68
	*n*	*%*	*n*	*%*	*χ*^*2*^	*df*	*p*-value
Parental marital status (divorced)	46	25.1	13	20.6	0.5	1	0.47
Family income (monthly)*					2.6	3	0.45
<1000	86	47.0	37	58.7			
1000–1999	70	38.3	19	30.2			
2000–3499	18	9.8	5	7.9			
>3500	9	4.9	2	3.2			

*: RMB (Chinese yuan)|:minimum monthly salary (per person).

### Predictive validity of the SAVRY total, protective and risk domain scores for violent reoffending

In the univariate logistic regression analysis, the Historical (OR = 1.1, 95% CI = 1.0–1.3) and Individual (OR = 1.2, 95% CI = 1.0–1.4) domains were significantly associated with increased risk of violent reoffending over the five year follow-up period (all p values<0.05; Table 2). In the ROC analysis, the Areas Under the Curve (AUCs) for the SAVRY Total Score, Protective Score, and the Historical, Social/Contextual, and Individual/Clinical Risk Domains ranged from 0.60 (for the SAVRY Protective Domain) to 0.68 (for the SAVRY Total Score) (Table 2).

**Table 2:** SAVRY total, domains scores, numbers of high risk items and the prediction of reoffending.

			Univariate logistic regression			Re-offending	
	M	sd	Exp(B)	Exp(B) 95% CI	*p*	AUC (SE)	95% CI
SAVRY Score							
Total Score	19.1	7.0				0.68 (0.04)	0.61, 0.76
Historical	7.4	3.3	1.1	1.0–1.3	0.04	0.66(0.04)	0.58, 0.74
Social/contextual	5.2	2.1	1.0	0.8–1.2	0.93	0.61(0.04)	0.53, 0.69
Individual	3.5	2.5	1.2	1.0–1.4	0.02	0.66 (0.04)	0.59, 0.74
Protective	2.9	1.6	1.2	1.0–1.5	0.07	0.60 (0.04)	0.52, 0.68
Numbers of high risk items							
Historical	2.1	1.6	1.3	1.0–1.7	0.06		
Social/contextual	1.3	1.2	1.0	0.7–1.5	0.82		
Individual	1.5	1.5	1.4	1.0–1.9	0.02		
Protective	2.9	1.6	0.9	0.7–1.2	0.61		

Note: AUC:area under the curve; CI:confidence interval.

Cronbach’s alpha ranged between 0.68 for the SAVRY Protective Domain to 0.89 for the SAVRY Total Score suggesting that these scales were associated with a high degree of internal consistency in this sample.

### Association between number of high-risk items on the SAVRY and violent reoffending

The univariate logistic regression analysis suggested that the number of high-risk items in the Individual/Clinical Risk Domains was significantly associated with an increased risk of violent reoffending over the five year follow-up period (OR = 1.4, 95% CI = 1.0–1.9; p value 0.02; Table 2). The number of high risk items on the Historical, Social/Contextural, and Protective Domains, however, were not significantly associated with an increased risk of violent reoffending in this sample (Table 2).

### Association between individual SAVRY items and violent reoffending

Univariate logistic regression analysis suggested that seven of the 30 individual SAVRY risk and protective items were significantly correlated with violent reoffending. Together these seven items were responsible for explaining 36.2% of the variance in violent reoffending in this sample. No protective items were significantly associated with violent reoffending in this sample, however.

Backward stepwise multiple logistic regression analyses suggested that only four items were independently predictive of violent recidivism: item 2 (‘history of non-violent offending’), item 17 (‘negative attitudes’), item 18 (‘risk-taking/impulsivity’), and item 20 (‘anger management problems’) (Table 3). These four items were together responsible for explaining 25.0% of the variance in violent reoffending in this sample (Table 3).

**Table 3:** SAVRY-items and prediction of reoffending.

Items of SAVRY	SAVRY中文条目	low	moderate	high	Univariate logistic regression			Stepwise backward regression		
					Exp(B)	Exp(B) 95% CI	p	Exp(B)	Exp(B) 95% CI	p
Historical risk factors	历史因子									
1.History of violence	1:既往暴力事件	24(9.8%)	60(24.4%)	162(65.9%)	0.8	0.4–1.7	0.54			
2.History of non-violent offending	2:非暴力犯罪史	98(39.8%)	69(28.0%)	79(32.1%)	2.5	1.5–4.3	<0.01	2.4	1.6–3.6	<0.01
3. Early initiation of violence	3.早年发生暴力事件	76(30.9%)	95(38.6%)	75(30.5%)	0.6	0.3–1.1	0.06			
4. Past supervision/intervention failures	4. 既往监管/干预失败	126(51.2%)	72(29.3%)	48(19.5%)	0.9	0.5–1.5	0.61			
5. History of self harm or suicide attempts	5. 既往自伤史或者曾企图自杀	217(88.2%)	24(9.8%)	5(2.0%)	1.6	0.7–3.8	0.30			
6. Exposure to violence in the home	6. 处于家庭暴力环境	105(42.7%)	113(45.9%)	28(11.4%)	1.0	0.6–1.9	0.97			
7. Childhood history of maltreatment	7. 童年受虐史	109(44.3%)	114(46.3%)	23(9.3%)	1.6	0.8–3.1	0.20			
8. Parental/caregiver criminality	8.父母/照料者存在犯罪史	197(80.1%)	37(15.0%)	12(4.9%)	1.7	0.8–3.3	0.14			
9. Early caregiver disruption	9.早期照料者分离	139(56.5%)	63(25.6%)	44(17.9%)	0.7	0.4–1.1	0.13			
10. Poor school achievement	10.在校期间,学业成就差	70(28.5%)	125(50.8%)	51(20.7%)	1.2	0.7–2.1	0.59			
Social/contextual risk factors	社会/环境因子									
11. Peer delinquency	11.同伴违纪	26(10.6%)	89(36.2%)	131(53.3%)	0.8	0.4–1.6	0.52			
12. Peer rejection	12.同伴排斥	141(57.3%)	94(38.2%)	11(4.5%)	1.3	0.7–2.5	0.44			
13. Stress and poor coping	13.应激和应对不当	147(59.8%)	85(34.6%)	14(5.7%)	0.6	0.3–1.3	0.20			
14. Poor parental management	14.父母监管不力	50(20.3%)	123(50.0%)	73(29.7%)	1.7	0.9–3.4	0.13			
15. Lack of personal/social support	15.缺乏他人/社会支持	70(28.5%)	121(49.2%)	55(22.4%)	0.6	0.3–1.1	0.09			
16. Community disorganization	16.社区治安混乱	72(29.3%)	137(55.7%)	37(15.0%)	2.0	1.0–3.7	0.04			
Individual risk factors	自身/临床因子									
17. Negative attitudes	17.消极敌视态度	62(25.2%)	145(58.9%)	39(15.9%)	2.8	1.4–5.5	<0.01	2.4	1.4–4.3	<0.01
18. Risk taking/Impulsivity	18.喜欢冒险/冲动	48(19.5%)	138(56.1%)	60(24.4%)	1.8	1.0–3.3	0.06	1.9	1.1–3.2	0.02
19. Substance use difficulties	19.物质滥用问题	134(54.5%)	103(41.9%)	9(3.7%)	0.9	0.3–1.2	0.13			
20. Anger management problems	20.难以调控愤怒情绪	113(45.9%)	112(45.5%)	21(8.5%)	1.8	1.0–3.3	0.07	1.7	1.0–2.8	0.04
21. Psychopathic traits (low empathy/remorse)	21.情感肤浅/缺乏同情心	94(38.2%)	123(50.0%)	29(11.8%)	0.9	0.5–1.6	0.63			
22.Attention-deficit/hyperactivity difficulties	22.注意缺陷/多动障碍	128(52.0%)	94(38.2%)	24(9.8%)	1.1	0.6–1.9	0.82			
23. Poor compliance	23.依从性差	77(31.3%)	109(44.3%)	60(24.4%)	1.1	0.6–2.1	0.69			
24. Low interest/Commitment to school	24.学习兴趣较低/不想上学	48(19.5%)	69(28.0%)	129(52.4%)	1.4	0.8–2.4	0.23			
Protective factors	保护因子	No		Yes						
1. Prosocial involvement	1.参与社会化活动	161(65.4%)		85(34.6%)	0.8	0.3–1.8	0.56			
2. Strong social support	2.强大的社会支持系统	104(42.3%)		142(57.7%)	1.0	0.4–2.3	0.93			
3. Strong attachment and bonds	3.强大可依靠的同盟网络	140(56.9%)		106(43.1%)	1.2	0.5–2.7	0.68			
4. Positive attitude toward intervention and authority	4.对干预和权威抱积极的态度	125(50.8%)		121(49.2%)	1.1	0.5–2.5	0.83			
5. Strong commitment to school	5.强烈的上学意愿	66(26.8%)		180(73.2%)	0.8	0.3–2.1	0.72			
6. Resilient personality traits	6.灵活有弹性的人格特征	155(63.0%)		91(37.0%)	1.3	0.6–3.0	0.53			

## Discussion

Overall, we found that average scores on each of the individual SAVRY risk or protective items were lower in this sample compared to Western samples [20–24]. Nevertheless, over an average follow-up period of five years, ROC analyses suggested that the SAVRY Risk Domains were predictive of violent reoffending in male juvenile offenders in China (AUCs: Total Score: 0.68; Historical Domain: 0.66; Social/Contextual Domain: 0.61; Individual/Clinical Domain: 0.66; Protective Domain: 0.60).

The predictive value of the Protective Domain was significantly higher in this study, as compared to other studies in European or North American samples [20,21], whilst the Individual Risk Domain appeared to be associated with lower predictive validity in this sample [21,23,25,26]. The predictive value of these domains is comparable to that found in a recent study of juvenile violent offenders in Singapore [14], although unlike this study, we found that the SAVRY Protective Domain was significantly associated with desistence from violent reoffending in this sample.

The AUCs of the various risk and protective domains as assessed by the SAVRY ranged from 0.60 to 0.70 in this study; slightly lower than those typically observed for a number of Western-developed actuarial and SPJ tools [27]. This suggests that additional risk factors unique to Chinese juvenile offenders may not be adequately captured by these tools at present [9]. Previous work with Chinese juvenile offenders, for example, suggests that although some factors protective against offending are similar to those captured by the SAVRY (e.g., strong commitment to school; strong social support) others may have unique importance for Chinese juveniles (e.g., an interest in social reform and culture) [28]. The valence of these items may not be adequately captured by existing items, derived from work with Western samples, assessing prosocial involvement.

To investigate which item/s might be associated with incremental predictive validity for the prediction of violence in juvenile Chinese offenders, we also investigated which item/s were independently associated with violence. Items on the Individual/Clinical Domain in particular appeared to be independently associated with the prediction of violence in this sample, including: item 17 (‘negative attitudes’), item 18 (‘risk-taking/impulsivity’), and item 20 (‘anger management problems’). Given that items assessed by the Individual/Clinical Domain are dynamic, and therefore are modifiable through planned interventions, results from the present study suggest that these factors in particular may be important treatment targets.

We found no particularly strong association between individual items on the Social/Contextural Domain or on the Protective Domain and violent reoffending in this sample. However, few of the offenders in our sample endorsed any of the protective items listed on the SAVRY (n = 14, 5.7%).

Additionally, none of the individual risk items on the SAVRY Protective Domain appeared to be independently predictive of violence in this population, including: prosocial involvement, strong social support, strong attachment and bonds, positive attitude toward intervention and authority, strong commitment to school, and resilient personality traits. There is therefore a need to determine whether protective factors associated with desistence from violence can be expected to translate across cultures [29].

### Limitations

This study has some limitations. First, as forensic psychiatrists in China currently do not use structured clinical judgement instruments to derive the SAVRY Summary Risk Rating, this information was typically not recorded in offenders’ file records. We were therefore unable to investigate the association between the Summary Risk Rating and the actual probability of violent reoffending in this study. The question therefore remains as to whether SPJ risk assessment tools are more accurate than actuarial risk assessment tools or unstructured clinical judgement in China.

Second, as the relative importance and/or predictive validity of risk and protective factors may differ between males and females, results from the present study cannot be used to inform the assessment of violence risk in female offenders presently. Further work is required to assess the performance of the SAVRY in both female offenders, as well as in non-detained samples.

Third, some psychiatric disorders, such as schizophrenia and other psychoses, bipolar disorder, and personality disorder have been found to contribute to increased violent reoffending risk in males, even following adjustment for prior offending [30]. Few studies have investigated the utility of the SAVRY for the prediction of violent reoffending in juvenile forensic psychiatric populations, however. Future studies should therefore consider the predictive validity of the SAVRY in these groups, particularly given research suggesting that some violence risk assessment instruments may be associated with lower predictive validity estimates when used to previous violence risk in samples with schizophrenia [31].

Fourth, information on violent reoffending at follow-up was collected from official police records. Therefore, only offences that were detected and/or reported to police will have been included. However, as most juvenile offenders are subject to parole orders, and are therefore supervised by the police in the community on their release from the YDCs in China, detection bias is likely to have been minimal. Reporting bias, however, cannot be ruled out.

Last, predictive validity is a function of both discrimination (i.e., the ability of a given violence risk assessment tool to distinguish between those who went on to violently re-offend from those who did not violently reoffend) and calibration (i.e., how well the prediction of violence risk made by a given violence risk assessment instrument concurs with actual observed risk) [32]. The AUC, however, measures only the former aspect. We therefore recommend that, in addition to measures of discrimination, future research into the predictive value of the SAVRY should report measures of calibration including sensitivity, specificity, positive predictive values (PPVs), negative predictive values (NPV), number needed to detain (NND) and number safely released (NSR) [33].

### Conclusions

Results from the present study would suggest that the SAVRY can be meaningfully used in China as a basis for assisting mental health, criminal justice, and juvenile probation officers, as well as policy makers, to develop, implement, and evaluate effective assessment and management approaches for male juvenile offenders. The SAVRY should not be used to justify custodial, security, or release decisions in China presently, however, owing to the relatively low predictive performance of many of the individual risk and protective factors assessed.

## Supporting Information

S1 File This is the raw data of the present study. (SAV)
